# Cementless Titanium Mesh Fixation of Osteoporotic Burst Fractures of the Lumbar Spine Leads to Bony Healing: Results of an Experimental Sheep Model

**DOI:** 10.1155/2016/4094161

**Published:** 2016-02-25

**Authors:** Anica Eschler, Paula Roepenack, Jan Roesner, Philipp Karl Ewald Herlyn, Heiner Martin, Martin Reichel, Robert Rotter, Brigitte Vollmar, Thomas Mittlmeier, Georg Gradl

**Affiliations:** ^1^Department of Trauma, Hand and Reconstructive Surgery, University of Rostock, Medical Center, Schillingallee 35, 18057 Rostock, Germany; ^2^Clinic for Anesthesiology and Critical Care Medicine, University of Rostock, Medical Center, Schillingallee 35, 18059 Rostock, Germany; ^3^Institute for Biomedical Engineering, University of Rostock, F. Barnewitz-Straße 4, 18119 Rostock, Germany; ^4^Faculty of Mechanical Engineering and Marine Technology, Chair of Mechanical Engineering Design/Lightweight Design, University of Rostock, Albert-Einstein-Straße 2, 18059 Rostock, Germany; ^5^Rudolf-Zenker Institute for Experimental Surgery, University of Rostock, Medical Center, Schillingallee 69a, 18057 Rostock, Germany; ^6^Department of Trauma, Orthopedic and Reconstructive Surgery, Munich Municipal Hospital Group, Clinic Harlaching, Sanatoriumsplatz 2, 81545 Munich, Germany

## Abstract

*Introduction*. Current treatment strategies for osteoporotic vertebral compression fractures (VCFs) focus on cement-associated solutions. Complications associated with cement application are leakage, embolism, adjacent fractures, and compromise in bony healing. This study comprises a validated VCF model in osteoporotic sheep in order to (1) evaluate a new cementless fracture fixation technique using titanium mesh implants (TMIs) and (2) demonstrate the healing capabilities in osteoporotic VCFs.* Methods*. Twelve 5-year-old Merino sheep received ovariectomy, corticosteroid injections, and a calcium/phosphorus/vitamin D-deficient diet for osteoporosis induction. Standardized VCFs (type AO A3.1) were created, reduced, and fixed using intravertebral TMIs. Randomly additional autologous spongiosa grafting (G1) or no augmentation was performed (G2, *n* = 6 each). Two months postoperatively, macroscopic, micro-CT and biomechanical evaluation assessed bony consolidation.* Results*. Fracture reduction succeeded in all cases without intraoperative complications. Bony consolidation was proven for all cases with increased amounts of callus development for G2 (58.3%). Micro-CT revealed cage integration. Neither group showed improved results with biomechanical testing.* Conclusions*. Fracture reduction/fixation using TMIs without cement in osteoporotic sheep lumbar VCF resulted in bony fracture healing. Intravertebral application of autologous spongiosa showed no beneficial effects. The technique is now available for clinical use; thus, it offers an opportunity to abandon cement-associated complications.

## 1. Introduction

In osteoporosis rarefication and thinning of intrinsic bone structures leads to reduced bone quality with inferior biomechanical properties [1, 2]. Vertebral compression fractures (VCFs) after low-velocity trauma currently occur with increasing numbers due to demographic age pattern changes [1, 3]. VCFs predominantly affect the thoracolumbar levels, with their high level of degrees of motion making implant fixation strength of particular interest; this is because internal fixation devices are likely to break out of or displace in osteoporotic bone [4]. Therefore cement-based treatment procedures, such as kyphoplasty or vertebroplasty techniques used alone or in combination with internal fixation, play a major role in this age group regardless of the fracture severity [5–11]. Using kyphoplasty or vertebroplasty an instant pain relief is achieved by transpedicular polymethylmethacrylate cement (PMMA) application [8–12]. Disadvantageous of intravertebral applied PMMA cement is the change of vertebral elasticity parameters, which results in a change in biomechanical properties that increases the likelihood of adjacent fractures (reported incidence: 8–26%) [3, 7, 9, 13–15]. In addition, complications such as pulmonary embolism (up to 20%), cement leakage into the epidural space, or injury of adjunct neurovascular structures can occur due to exothermic reactions during the polymerization process (4–13%) [5, 7, 10, 11, 15]. Apart from those complications, fracture treatment using cement depends upon bone-cement compound-fixation while disabling bony healing capacity of the fracture.

Astonishingly, VCF treatment modalities promoting fracture healing currently have no value in clinical practice. This finding is contrary to the customary clinical practice that provides osteoporotic fracture healing capabilities for other sites such as the proximal femur or distal radius [16, 17]. The question arises: what would be the ideal treatment modality to avoid the aforementioned problems? Theoretically, in order to promote bony healing, this could be a minimally invasive placed device (e.g., cage) that could provide intravertebral reduction while avoiding cement use. Recently developed intravertebral reduction systems enable this intravertebral reduction with reduced amounts of PMMA; however, they are not designated for cementless use [14, 18, 19]. Their use has proven reduction of cement-associated complications in initial clinical studies; anyhow their annihilation did not succeed [18, 20].

In order to enable experimental improvements and new treatment techniques for VCF, a workable spinal fracture model for creation of VCFs in osteoporotic Merino sheep was developed in a prior study [21]. Weekly corticosteroid therapy and a calcium/phosphorus/vitamin D-deficient diet was administered for 5.5 months following ovariectomy and severe osteoporosis of the lumbar spine was demonstrated by pQCT and micro-CT analyses. Creation of a VCF type A3.1 succeeded via an ALIF (Anterior Lumbar Interbody Fusion) approach and standardized compression method. Using this fracture model, it was hypothesized that (1) lumbar VCFs type A3.1 in osteoporotic sheep can be adequately restored using titanium mesh cages without cement application; (2) osteoporotic VCFs have bony healing capabilities that can be proven via micro-CT analyses; and (3) additional application of autologous spongiosa graft accelerates fracture healing with superior biomechanical properties.

## 2. Materials and Methods 

### 2.1. Ethics Statement

This randomized, controlled study was conducted in strict accordance with the European Union Legislation for the protection of animals used for scientific purposes and approved by the state's Animal Ethics Committee (Landesamt für Landwirtschaft, Lebensmittelsicherheit und Fischerei Mecklenburg-Vorpommern, Rostock, Germany; permit number 7221.3-1.1-007/13).

### 2.2. Animal Preparation, Surgical Procedure, and Randomization

For osteoporosis induction twelve 5-year-old female Merino sheep (mean body weight: 66.3 ± 2.0 kg; range: 57–79) were ovariectomized bilaterally (OP1), housed in a shed protecting them from sunlight, and fed a calcium/phosphorus/vitamin D-deficient diet and were injected weekly with dexamethasone (1.3 mg/kg body weight plus 0.028 mL/kg body weight dexamethasone-sodium-phosphate) for 5.5 ± 0.1 months (range: 5-6). Micro-CT and pQCT analyses documented significant lowering of BMD (bone mineral density) values for the second lumbar vertebrae for this model [21].

Animal preparation was performed under general anesthesia induced by 2.0 mg/kg body weight ketamine, 0.5 mg/kg body weight midazolam, and 0.010 mg/kg body weight fentanyl intravenously (IV) and maintained with 1.0–1.5% isoflurane and a FiO_2_ of 0.4 after 24 hours of fasting. Preoperatively, 0.1 mg/kg body weight xylazine was injected intramuscularly (IM) for sedation. Intra- and postoperatively, extreme effort was made to minimize suffering via the administration of perioperative analgesia with 2.5 g metamizole IV for the second half of the procedure and 0.005–0.010 mg/kg body weight fentanyl IV if the duration of the procedure exceeded 1 hour. The postoperative analgesia regimen included a combination of 25–50 mg/kg body weight metamizole IV/IM q6 h (later administered orally) and 0.005–0.010 mg/kg body weight fentanyl IV on demand. An IV prophylactic antibiotic (cefuroxime) was administered intraoperatively and postoperatively; 1 g menbutone IM was administered to promote absorption.

**Figure 1:**
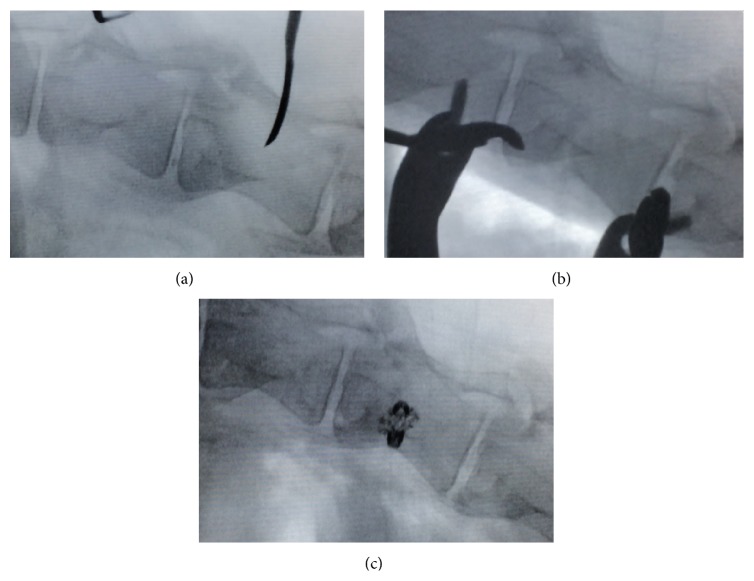
Intraoperative radiographic imaging preoperative (a), after fracture creation (b), and postinterventional VCF fixation by extrapedicular placement of the titanium mesh cage (c).

A standard minilumbotomy of 4-5 cm and ALIF approach to the second lumbar vertebra was performed in the left lateral position. Creation of a type A3.1 VCF of the second lumbar vertebral body was achieved in a standardized manner in all cases and resulted in a significant decrease in body angle and vertebral height as previously reported [21]. Immediate fracture care (OP2) was performed by extrapedicular placement of one intervertebral mesh cage (OsseoFix®, Alphatec Spine, Carlsbad, California, USA) for fracture reduction and maintenance of reduction. The mesh cage (4.5 mm) was deployed according to the manufacturer's recommendations, using the specific implant insertion devices while referencing intraoperative fluoroscopic imaging in anterior-posterior and lateral views. The implant was deployed until the sagittal index (SI) and vertebral body kyphotic angle (KA) as measures of segmental kyphosis at the level of the spine segment were adequately reduced (Figure 1). PMMA cement was not applied. Randomly, additional intravertebral autologous iliac crest spongiosa graft application (G2) or no additional augmentation (G1) was performed (*n* = 6 each). For G2, approximately 2 g autologous iliac crest spongiosa graft was acquired from a 1.5 cm incision on the right iliac crest and applied in the cage via the manufacturer's implant inserter cannula. After removal of instruments the surgical site was closed and immediate mobilization in an all-fours position was made.

The calcium/phosphorus/vitamin D-deficient diet was continued until euthanization was planned (2.2 ± 0.1 months; range: 2-2 after OP2) using IV propofol/T61 and the lumbar spine harvested immediately after death.

### 2.3. Macroscopic Analysis

The former fracture line and amount of callus development was assessed after death. The circumference area 0.5 cm below the upper second lumbar vertebrae endplate was compared to the third lumbar vertebrae circumference via analogue localization.

### 2.4. High-Resolution Three-Dimensional (3D) Micro-CT and pQCT Analysis

For quantitative and 3D micro-CT analysis a Skyscan 1076 micro-CT scanner and software package for reconstruction and analysis (CTAn, V1.1; NRecon, Version 1.6.6.0; Bruker Corporation, Billerica, MA, USA) was used according to manufacturer's recommendations (Method note: Phil Salmon, Bruker Inc.). The former fracture region of the second lumbar was analyzed for callus formation. A group of 7 healthy sheep and 4 osteoporotic sheep was used as control in order to compare nonosteoporotic to osteoporotic bone structural parameter. After 12 hours in 0.9% saline, each vertebra was wrapped in parafilm and placed in a polypropylene tube to prevent drying and associated motion artefacts during scanning. A foam tube allowed horizontal mounting in the sample chamber for *μ*CT imaging. With this approach, all samples were oriented with the longitudinal axis parallel to the optical axis of the instrument. To determine BMD specimens with known mineral density and adequate size (bone phantoms) were assessed under the same conditions. Projection images were analyzed at 49 kV with 200 *μ*A X-ray intensity using a 0.5 mm aluminum beam-flattening filter. Spatial resolution was 18 *μ*m and the rotation step was 0.4°. Image reconstruction was performed with beam hardening correction of 30%, defect pixel masking of 20%, ring artifact reduction of 6, and an individual alignment correction calculated by the software. The reconstructed volume was oriented along the long axis of the vertebra and the region of interest covering trabecular bone tissue was drawn using a standardized circular region of interest for L3 and manually drawn region of interest for L2; this was done to diminish artifacts caused by the implanted cage. From the upper vertebral endplate, the first slice containing only cancellous bone was selected and then 101 lower slices were selected. Regions with artifacts or cortical bone were excluded. The volume of interest represented 178.78 ± 19.19 *μ*m^3^ (range: 93–298 *μ*m^3^) trabecular bone for L3 and 34.51 ± 1.78 *μ*m^3^ (range: 25–51 *μ*m^3^) callus formation for L2.

Three-dimensional reconstruction of the series of images visualized the degree of osseous integration of the cages and the proportion of the segmented bone volume relative to the total volume of interest (BV/TV), the average number of trabeculae per unit length (Tb.N), the mean thickness of trabeculae (Tb.Th), the mean distance between the trabeculae (Tb.Sp), and the structure model index (SMI). In order to estimate the precision of the analysis, the *μ*CT scans were analyzed independently three times by the same investigator.

Osteoporosis induction and retention were evaluated by changes in BMD using peripheral quantitative computed tomography (pQCT) of the right distal radius according to World Health Organization (WHO) osteoporosis guidelines [22, 23]. PQCT of the right distal radius was conducted for fracture creation/treatment as well as postmortem using a pQCT scanner (Stratec Medizintechnik, Pforzheim, Germany). An untreated group of sheep (7 slaughtered 5-year-old Merino sheep) served as control since in vivo pQCT-BMD analysis of the lumbar spine is not possible. In order to increase precision, three scans were performed and analyzed independently by the same investigator.

### 2.5. Biomechanical Analysis

**Figure 2:**
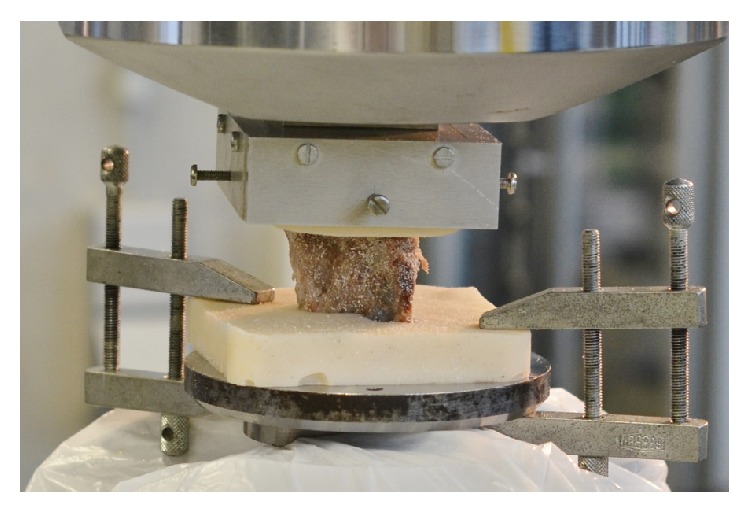
Experimental setup for mechanical testing. L2 vertebra is embedded with a two-component adhesive, placed in holding devices, fixed in hydraulic clamps, and loaded by a pressure plate on the upper vertebral body end plate.

A servohydraulic material testing machine type 322.21 (MTS, Eden Prairie, Minnesota, USA, 100 kN, MultiPurpose Testware, v 5.6A4585) was used in compression testing of lumbar vertebra L2 and 3 of each cadaver. Average failure load (*F*_max_) and stiffness (*c*_wk_) were calculated for each of the three groups for L2 and compared to the values of L3. Both the vertebrae's upper and lower endplates were embedded in a special two-component adhesive (Demotec 95; Nidderau, Germany) in order to ensure parallel application of force (Figure 2). In a displacement-controlled mode at a rate of 2 mm/min axial compression forces were applied between two parallel plates until macroscopic failure of the vertebra occurred or 16 kN was reached (at a data rate of 5 Hz). The load displacement diagram was documented and failure load was manually defined at the first significant decrease of slope; vertebral stiffness was defined from the first approximately linear range and the slope of the corresponding regression line. For a better comparability, data were calculated to a structural modulus (*E*_wk_) and limit stresses (*S*_wk_) according to their cross section area (*A*_wk_) and height (*h*_wk_) (*E*_wk_ = *c*_wk_/*A*_wk_ · *h*_wk_; *S*_wk_ = *F*_max_/*A*_wk_).

### 2.6. Statistical Analysis

Results are presented as mean ± SEM (Standard error of mean; range). After satisfying the assumption of normality (Kolmogorov-Smirnov test), the paired* t*-test analyses or the Mann-Whitney* U* Test (nonnormal distribution) was performed to analyze the differences in both groups. Significance was defined at *P* < 0.05. Statistical analysis was performed using SPSS Statistics version 20.0 software (IBM, Armonk, New York, USA).

## 3. Results

### 3.1. Fracture Healing

**Figure 3:**
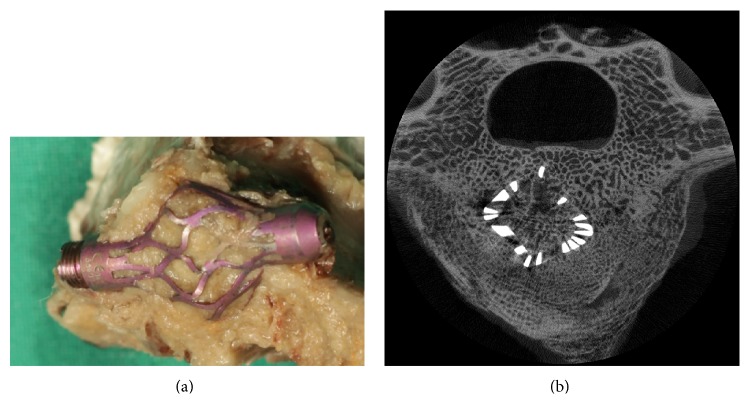
Macroscopic (a) and micro-CT reconstruction (b) displaying bony fracture healing with cage integration.

Macroscopic analyses revealed fracture healing in all cases (Figure 3(a)). The degree of fracture healing was assessed by evaluation of the fracture line and amount of callus development. The former fracture line showed bony consolidation in all cases. Four vertebrae (33.3%) showed an increased callus development with more than 1.5 cm increase in vertebral circumference (G1 *n* = 2; G2 *n* = 2). This callus development resulted in a 58.3% increase in mean vertebral circumference difference for the group with additional intravertebral autologous spongiosa grafting (G2 1.1 ± 0.4 cm, range: 1-2) when compared to G1 (0.6 ± 0.3 cm, range: 0-1).

Three-dimensional micro-CT analyses imaged the developed amorphous bone structure, henceforth referred to as callus, as well as cage integration. Cage integration with bony consolidation was revealed for all cases (Figure 3(b)). Histomorphometric micro-CT data showed a significant increase in BV/TV with 68.96 ± 4.69% (range: 35–86) when compared to osteoporotic (28.83 ± 2.29%, range: 23–33; *P* < 0.01) and healthy values (34.72 ± 2.54%; range: 27–48; *P* < 0.01); no differences were found between both treatment groups.  Table 1 outlines further histomorphometric parameters of the callus.

**Figure 4:**
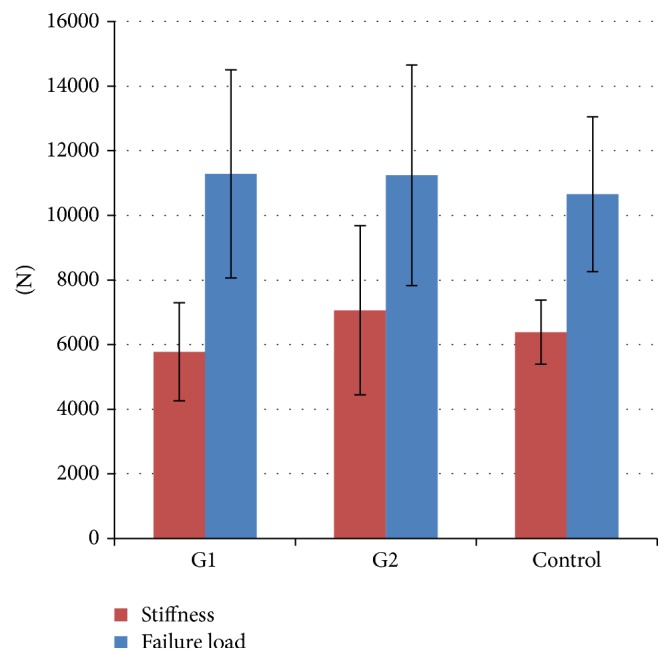
Biomechanical data. Vertebral failure load and stiffness in the treatment groups and unfractured osteoporotic vertebrae (control).

Biomechanical testing revealed similar vertebral failure load and vertebral stiffness when compared to unfractured osteoporotic vertebrae, thus confirming fracture healing in all cases (Figure 4). Additional autologous spongiosa application with the shown distinct callus development did not result in an increase in vertebral failure load or vertebral stiffness. After conversion to structural model/limit stresses according to their specific area/height, a slight tendency towards reduced normalized stiffness in G1 (302.13 ± 37.45 N/mm^2^; range: 163–393) was seen when compared to G2 (319.36 ± 39.63 N/mm^2^; range: 206–446) which was not significant; moreover, normalized failure load did not confirm that impression (G1 22.64 ± 4.35 N/mm^2^, range: 19–28; G2 24.02 ± 5.65 N/mm^2^, range: 15–32).

### 3.2. General Observations and Surgical Procedure

A mean weight gain of 2.1 ± 1.0 kg (range: −2 to +8; G1: 0.5 ± 1.0 kg, range: −2 to +4; G2: 3.7 ± 1.4 kg, range: 0 to +8) was observed before euthanization; this compensated for the mean weight loss of 3.2 ± 1.6 kg (range: −11 to +10) during deficient nutrition and the 5.5-month steroid therapy preoperatively to OP2.

Persistent osteoporosis was documented via pQCT-BMD measurement of the distal radius. 5.5 months after ovariectomy, weekly corticosteroid therapy, and a calcium/phosphorus/vitamin D-deficient diet the mean BMD was significantly lower (186.40 ± 6.69 mg/cm^3^; range: 133–216; *P* < 0.01) and did not differ between both groups (G1: 191.8 ± 8.29 mg/cm^3^, range: 162–216; G2: 181.00 ± 10.81 mg/cm^3^, range: 132–206), when compared to the control group (269.28 ± 11.37 mg/cm^3^; range: 230–318, SD ±34.13). For planned euthanization, BMD was persistently and significantly lower, thus revealing persistent osteoporotic bone (181.59 ± 7.75 mg/cm^3^; range: 131–225; *P* < 0.01; G1: 170.28 ± 11.99 mg/cm^3^, range: 131–210; G2: 192.90 ± 8.32 mg/cm^3^, range: 170–225).

Fracture care of the L2 VCF type A3.1 succeeded in all cases without intraoperative or postoperative complications. Mean anesthesia time for the procedures (including fracture creation) was 137.1 ± 7.2 minutes (range: 110–180; G1: 142.5 ± 9.3 minutes, range: 120–180; G2: 131.7 ± 11.4 minutes, range: 110–180).

In regard to body angle or vertebral height in lateral X-ray views, fracture creation resulted in an increase of KA of 4.1 ± 0.8° (range: 0–11), while SI increased by 4.5 ± 1.0% (range: 2–12; Table 2). With consecutive fracture care using the intravertebral titanium mesh cage adequate vertebral body reduction was achieved in all cases: KA decreased by a mean of 3.8 ± 0.5° (range: 2–7). Correspondingly, SI decreased by 4.1 ± 1.1% (range: 0–11). All surgical incisions healed uneventfully.

## 4. Discussion

Fracture reduction and fixation using titanium mesh implants in a validated sheep model of osteoporotic lumbar spine incomplete burst fractures (A3.1) result in bony fracture healing without cement application. As hypothesized, bony healing was proven with macroscopic, micro-CT, and biomechanical evaluation. Additional intravertebral application of autologous spongiosa leads to increased callus formation; however, without superior biomechanical properties.

Reports of fracture models in animals, which are able to experimentally reproduce a specific fracture type that frequently occurs in humans, are rare; to date, only studies of bone defect models have been published [24, 25]. In addition, studies of this nature have not been conducted on healthy vertebrae [26–30]. Sheep spines have been demonstrated to be biomechanically similar to human spines with similar loads; however, higher axial compression stresses concomitant with slightly higher bone densities are due to quadrupedal locomotion [31]. Multiple studies have demonstrated the sheep's usefulness for osteoporosis research and bone healing due to their genetical similarity to humans with their estrus cycle, hormone profiles, and Harversian bone remodeling [32–36]. A current literature review of Sheng et al. [37] found the sheep spine as the most useful experimental in vivo model of the lumbar spine when compared to human anatomical parameters involving cost and availability thoughts. Furthermore, the comparison of micro-CT structural changes of the vertebral cancellous bone in between our results and human data indicates that a similar osteoporosis degree to that in the clinical situation was induced in our sheep. Due to these similarities to the human bone remodeling and human spine, this study comprised a previously published validated osteoporotic VCF sheep model.

For VCF surgery, it is well known that osteoporosis due to decreased BMD affects the implant-bone interface, leading to inferior biomechanical properties [27]. Thus, cement-associated solutions such as vertebroplasty or kyphoplasty techniques are the gold standard for clinical practice; however, they are associated with the specific aforementioned complications [5–15, 19, 27]. In order to diminish these cement-related complications, this study compromised a specific titanium mesh implant designated for cementless application (currently, an off-label use) in 12 osteoporotic sheep with unstable burst VCFs. Vertebral body reduction succeeded in all cases. These reduction capabilities concur with a few previously published biomechanical studies of the titanium mesh implant: Upasani et al. [38] compared the implants to standard balloon kyphoplasty and reported a significantly greater height maintenance as well as decreased cement amounts. Ghofrani et al. [14] reported similar results in human spine cadavers that spared additional cement application. The first subsequent clinical trial revealed improvements in pain (Visual analogue scale 7.7 to 1.4), Oswestry-Disability Index (71% to 30%), and adequate reduction (Cobb angle 11.7° to 10.4°) in a 12-month follow-up of 32 osteoporotic VCFs stabilized with the titanium mesh cage; however, PMMA cement was still used [20]. Admittedly, reduction properties in our study do not fully match the situation in humans since surgical resolution of osteoporotic spine fractures without neurologic impairments is rarely performed immediately after the injury. This denoted a study limitation because clinical experience indicates a more difficult fracture reduction with delayed surgery. Nevertheless, one first published clinical trial presented preliminary results in four patients with off-label cementless use of the titanium mesh cage and revealed similarly adequate fracture reduction (KA 14.5° to 10.7°; Cobb 10.1° to 8.3°) [18].

In addition to the adequate reduction capabilities of the implant, the cementless procedure demonstrated bony healing of the osteoporotic VCFs two months postoperatively. Although it is well documented that patients suffering from osteoporosis have an increased fracture risk, and prevention methods are well established, the properties of osteoporotic fracture healing have received little attention [17, 39, 40]. In contrast, current clinical practice with kyphoplasty/vertebroplasty techniques leading the way disables bony healing capacity via intravertebral cement application. Recent animal studies in rats and sheep confirm fracture healing capabilities under low BMD conditions, and interestingly both decreased [41–43] as well as increased callus formation [44] was observed. In addition, clinical trials in humans confirm healing capabilities in osteoporotic bone, although they reveal an abnormality of bone remodeling [16, 17]. Osteoporosis fracture healing capabilities can be explained by the different pathways of bone remodeling resembling different aspects of embryological skeletal progresses [45].

In addition to the aforementioned fracture healing impairments, low BMD-associated problems such as implant loosening or breakage have been reported, which might be reduced by an enhancement of fracture repair. This was aimed at with additional intravertebral autologous iliac crest spongiosa graft application for one group. Anyhow, despite the distinct increased amount of callus, we observed no superior biomechanical properties. This concurs with other studies [46]; for example, Peter et al. [47] described an alendronate-induced increase in callus size in dogs that had undergone a radius fracture, without an accompanying improvement in flexural rigidity or failure-load. This might be due to the fact that the rapidly induced increase in callus size leads to an early gain of strength (strength, resp., stiffness is related to the third, resp., fourth power of the radius), while bone remodeling in osteoporosis is delayed; consequently bone formation rates and mineral apposition are reduced [48]. Thus, mechanical integrity of the callus in this early stage might be impaired and a time-dependent coefficient may be implied as other studies have revealed improved mechanical strength after 6 months in similar models [24]. Therefore, our data do not reveal any advantage to additional spongiosa graft application. Moreover, a lack of biomechanical data after the course of 2 months is a limitation of the study, coming with the study design.

Further study limitations in terms of bony healing include a certain inherent subjectivity bias that occurs with the macroscopic evaluation of the fracture gap. In clinical practice healing is defined as combined clinical and radiological findings, whereas radiological evaluation is difficult to assess [49]. Eventually, the biomechanically demonstrated stability proves bony healing.

## 5. Conclusions

Our study results indicate that fracture fixation using the titanium mesh implant provides a sound mechanical basis for fracture reduction and healing even in osteoporotic bone. Thus, cement-associated complications can be avoided. Prospective clinical trials are needed to determine its value for clinical use.

## Figures and Tables

**Table 1:** Micro-CT histomorphometric 3D parameters of healthy bone (T0), osteoporotic bone (T1), the callus formation (T2), and its differences in both treatment groups.

Time point	BV/TV (%)	SMI	Tb.N (mm^−1^)	Tb.Th (mm)	Tb.Sp (mm)
T0	34.72 ± 2.54 (27.12–48.50)	0.15 ± 0.03 (0.04–0.29)	1.56 ± 0.05 (1.34–1.74)	0.22 ± 0.01 (0.18–0.28)	0.58 ± 0.04 (0.40–0.71)

T1	28.83 ± 2.29 (22.60–33.09)	0.14 ± 0.12 (0.07–0.39)	1.65 ± 0.12 (1.33–1.83)	0.17 ± 0.00 (0.17–0.18)^*∗*^	0.52 ± 0.01 (0.49–0.54)

T2	68.96 ± 4.69 (35.11–86.02)^*∗∗*^	−3.68 ± 0.74 (−7.33–+0.82)^*∗∗*^	2.49 ± 0.13 (2.09–3.60)^*∗∗*^	0.29 ± 0.03 (0.12–0.40)^*∗∗*^	0.20 ± 0.01 (0.14–0.25)^*∗∗*^

T2G1	71.65 ± 5.07 (51.81–79.82)	−2.56 ± 0.81 (−4.15–+0.45)	2.21 ± 0.06 (2.09–3.40)	0.33 ± 0.03 (0.26–0.40)	0.22 ± 0.01 (0.20–0.25)

T2G2	66.28 ± 7.80 (35.11–86.02)	−2.81 ± 1.25 (−7.33–+0.82)	2.77 ± 0.17 (2.41–3.60)	0.25 ± 0.04 (0.12–0.36)	0.18 ± 0.01 (0.14–0.22)

^*∗*^T1 versus T0 *P* < 0.05; ^*∗∗*^T2 versus T1 *P* < 0.01.

**Table 2:** Intraoperative vertebral body reduction in regard to body angle (KA) and vertebral height (SI) with changes in preoperative values to those after fracture creation (T1) and values after fracture creation to those postinterventional VCF fixations (T2).

	KA (°)		SI (%)	
	Mean ± SEM	Range	Mean ± SEM	Range
T1	Increase in value, healthy versus fracture creation			
G1 + 2	4.1 ± 0.8	1–10	4.5 ± 1.0	2–12
G1 No augmentation	4.2 ± 1.1	1–8	4.2 ± 1.6	2–11
G2 Spongiosa	3.9 ± 1.3	2–10	4.9 ± 1.6	3–12

T2	Decrease in value, fracture creation versus VCF fixation			
G1 + 2	3.8 ± 0.5	2–7	4.1 ± 1.1	0–11
G1 No augmentation	3.5 ± 0.9	1–7	4.3 ± 1.9	0–11
G2 Spongiosa	4.2 ± 0.7	3–7	3.9 ± 1.3	1–10

## References

[1] Goldhahn S., Kralinger F., Rikli D., Marent M., Goldhahn J. (2010). Does osteoporosis increase complication risk in surgical fracture treatment? A protocol combining new endpoints for two prospective multicentre open cohort studies. *BMC Musculoskeletal Disorders*.

[2] Mittra E., Rubin C., Qin Y.-X. (2005). Interrelationship of trabecular mechanical and microstructural properties in sheep trabecular bone. *Journal of Biomechanics*.

[3] Movrin I. (2012). Adjacent level fracture after osteoporotic vertebral compression fracture: a nonrandomized prospective study comparing balloon kyphoplasty with conservative therapy. *Wiener Klinische Wochenschrift*.

[4] Goldhahn J., Suhm N., Goldhahn S., Blauth M., Hanson B. (2008). Influence of osteoporosis on fracture fixation—a systematic literature review. *Osteoporosis International*.

[5] Walter J., Haciyakupoglu E., Waschke A., Kalff R., Ewald C. (2012). Cement leakage as a possible complication of balloon kyphoplasty—is there a difference between osteoporotic compression fractures (AO type A1) and incomplete burst fractures (AO type A3.1)?. *Acta Neurochirurgica*.

[6] Korovessis P., Vardakastanis K., Repantis T., Vitsas V. (2014). Transpedicular vertebral body augmentation reinforced with pedicle screw fixation in fresh traumatic A2 and A3 lumbar fractures: comparison between two devices and two bone cements. *European Journal of Orthopaedic Surgery and Traumatology*.

[7] Han S., Wan S., Ning L., Tong Y., Zhang J., Fan S. (2011). Percutaneous vertebroplasty versus balloon kyphoplasty for treatment of osteoporotic vertebral compression fracture: a meta-analysis of randomised and non-randomised controlled trials. *International Orthopaedics*.

[8] Lovi A., Teli M., Ortolina A., Costa F., Fornari M., Brayda-Bruno M. (2009). Vertebroplasty and kyphoplasty: complementary techniques for the treatment of painful osteoporotic vertebral compression fractures. A prospective non-randomised study on 154 patients. *European Spine Journal*.

[9] Zou J., Mei X., Zhu X., Shi Q., Yang H. (2012). The long-term incidence of subsequent vertebral body fracture after vertebral augmentation therapy: a systemic review and meta-analysis. *Pain Physician*.

[10] Omidi-Kashani F., Samini F., Hasankhani E. G., Kachooei A. R., Toosi K. Z., Golhasani-Keshtan F. (2013). Does percutaneous kyphoplasty have better functional outcome than vertebroplasty in single level osteoporotic compression fractures? A comparative prospective study. *Journal of Osteoporosis*.

[11] Hulme P. A., Krebs J., Ferguson S. J., Berlemann U. (2006). Vertebroplasty and kyphoplasty: a systematic review of 69 clinical studies. *Spine*.

[12] Lee H. M., Park S. Y., Lee S. H., Suh S. W., Hong J. Y. (2012). Comparative analysis of clinical outcomes in patients with osteoporotic vertebral compression fractures (OVCFs): conservative treatment versus balloon kyphoplasty. *Spine Journal*.

[13] Eom K. S., Kim T. Y. (2012). Percutaneous vertebroplasty-induced adjacent vertebral compression fracture. *Pain Physician*.

[14] Ghofrani H., Nunn T., Robertson C., Mahar A., Lee Y., Garfin S. (2010). An evaluation of fracture stabilization comparing kyphoplasty and titanium mesh repair techniques for vertebral compression fractures: is bone cement necessary?. *Spine*.

[15] Lin E. P., Ekholm S., Hiwatashi A., Westesson P.-L. (2004). Vertebroplasty: cement leakage into the disc increases the risk of new fracture of adjacent vertebral body. *American Journal of Neuroradiology*.

[16] Büyükkurt C. D., Bülbül M., Ayanoğlu S., Esenyel C. Z., Öztürk K., Gürbüz H. (2012). The effects of osteoporosis on functional outcome in patients with distal radius fracture treated with plate osteosynthesis. *Acta Orthopaedica et Traumatologica Turcica*.

[17] Hoang-Kim A., Gelsomini L., Luciani D., Moroni A., Giannini S. (2009). Fracture healing and drug therapies in osteoporosis. *Clinical Cases in Mineral and Bone Metabolism*.

[18] Eschler A., Ender S. A., Ulmar B., Herlyn P., Mittlmeier T., Gradl G. (2014). Cementless fixation of osteoporotic VCFs using titanium mesh implants (OsseoFix): preliminary results. *BioMed Research International*.

[19] Hartmann F., Griese M., Dietz S. O., Kuhn S., Rommens P. M., Gercek E. (2015). Two-year results of vertebral body stenting for the treatment of traumatic incomplete burst fractures. *Minimally Invasive Therapy & Allied Technologies*.

[20] Ender S. A., Wetterau E., Ender M., Kühn J.-P., Merk H. R., Kayser R. (2013). Percutaneous stabilization system Osseofix® for treatment of osteoporotic vertebral compression fractures—clinical and radiological results after 12 months. *PLoS ONE*.

[21] Eschler A., Röpenack P., Herlyn P. K. (2015). The standardized creation of a lumbar spine vertebral compression fracture in a sheep osteoporosis model induced by ovariectomy, corticosteroid therapy and calcium/phosphorus/vitamin D-deficient diet. *Injury*.

[22] World Health Organization (1994). Assessment of fracture risk and its implication to screening for postmenopausal osteoporosis. *Technical Report Series*.

[23] Osteologischen Gesellschaften e.V. (DVO) (2014). *S3-Leitlinie des Dachverbands der Deutschsprachigen Wissenschaftlichen Osteologischen Gesellschaften e.V. (DVO). Prophylaxe, Diagnostik und Therapie der Osteoporose*.

[24] Phillips F. M., Turner A. S., Seim H. B. (2006). In vivo BMP-7 (OP-1) enhancement of osteoporotic vertebral bodies in an ovine model. *Spine Journal*.

[25] Zhu X. S., Zhang Z. M., Mao H. Q. (2011). A novel sheep vertebral bone defect model for injectable bioactive vertebral augmentation materials. *Journal of Materials Science: Materials in Medicine*.

[26] Galovich L. A., Perez-Higueras A., Altonaga J. R., Orden J. M. G., Barba M. L. M., Morillo M. T. C. (2011). Biomechanical, histological and histomorphometric analyses of calcium phosphate cement compared to PMMA for vertebral augmentation in a validated animal model. *European Spine Journal*.

[27] Li Y., Cheng H., Liu Z.-C. (2013). In vivo study of pedicle screw augmentation using bioactive glass in osteoporosis sheep. *Journal of Spinal Disorders & Techniques*.

[28] Liu D., Zhang Y., Zhang B. (2013). Comparison of expansive pedicle screw and polymethylmethacrylate-augmented pedicle screw in osteoporotic sheep lumbar vertebrae: biomechanical and interfacial evaluations. *PLoS ONE*.

[29] Krijnen M. R., Mullender M. G., Smit T. H., Everts V., Wuisman P. I. J. M. (2006). Radiographic, histologic, and chemical evaluation of bioresorbable 70/30 poly-L-lactide-CO-D, L-lactide interbody fusion cages in a goat model. *Spine*.

[30] Mullender M. G., Krijnen M. R., Helder M. N., Smit T. H., Everts V., Wuisman P. I. J. M. (2007). Lumbar body fusion with a bioresorbable cage in a goat model is delayed by the use of a carboxymethylcellulose-stabilized collagenous rhOP-1 device. *Journal of Orthopaedic Research*.

[31] Wilke H.-J., Kettler A., Wenger K. H., Claes L. E. (1997). Anatomy of the sheep spine and its comparison to the human spine. *Anatomical Record*.

[32] Lill C. A., Lill C. A., Fluegel A. K., Schneider E. (2002). Effect of ovariectomy, malnutrition and glucocorticoid application on bone properties in sheep: a pilot study. *Osteoporosis International*.

[33] Zarrinkalam M. R., Beard H., Schultz C. G., Moore R. J. (2009). Validation of the sheep as a large animal model for the study of vertebral osteoporosis. *European Spine Journal*.

[34] Chavassieux P., Buffet A., Vergnaud P., Garnero P., Meunier P. J. (1997). Short-term effects of corticosteroids on trabecular bone remodeling in old ewes. *Bone*.

[35] Hornby S. B., Ford S. L., Mase C. A., Evans G. P. (1995). Skeletal changes in the ovariectomised ewe and subsequent response to treatment with 17*β* oestradiol. *Bone*.

[36] Kettler A., Liakos L., Haegele B., Wilke H.-J. (2007). Are the spines of calf, pig and sheep suitable models for pre-clinical implant tests?. *European Spine Journal*.

[37] Sheng S.-R., Wang X.-Y., Xu H.-Z., Zhu G.-Q., Zhou Y.-F. (2010). Anatomy of large animal spines and its comparison to the human spine: a systematic review. *European Spine Journal*.

[38] Upasani V. V., Robertson C., Lee D., Tomlinson T., Mahar A. T. (2010). Biomechanical comparison of kyphoplasty versus a titanium mesh implant with cement for stabilization of vertebral compression fractures. *Spine*.

[39] Goldhahn J., Little D., Mitchell P. (2010). Evidence for anti-osteoporosis therapy in acute fracture situations—recommendations of a multidisciplinary workshop of the International Society for Fracture Repair. *Bone*.

[40] Goldhahn J., Blauth M. (2007). Osteoporotic fracture management: closing the gap of knowledge. *Archives of Orthopaedic and Trauma Surgery*.

[41] Hao Y. J., Zhang G., Wang Y. S. (2007). Changes of microstructure and mineralized tissue in the middle and late phase of osteoporotic fracture healing in rats. *Bone*.

[42] Lill C. A., Hesseln J., Schlegel U., Eckhardt C., Goldhahn J., Schneider E. (2003). Biomechanical evaluation of healing in a non-critical defect in a large animal model of osteoporosis. *Journal of Orthopaedic Research*.

[43] Namkung-Matthai H., Appleyard R., Jansen J. (2001). Osteoporosis influences the early period of fracture healing in a rat osteoporotic model. *Bone*.

[44] Kubo T., Shiga T., Hashimoto J. (1999). Osteoporosis influences the late period of fracture healing in a rat model prepared by ovariectomy and low calcium diet. *Journal of Steroid Biochemistry and Molecular Biology*.

[45] Gerstenfeld L. C., Cullinane D. M., Barnes G. L., Graves D. T., Einhorn T. A. (2003). Fracture healing as a post-natal developmental process: molecular, spatial, and temporal aspects of its regulation. *Journal of Cellular Biochemistry*.

[46] Amanat N., Brown R., Bilston L. E., Little D. G. (2005). A single systemic dose of pamidronate improves bone mineral content and accelerates restoration of strength in a rat model of fracture repair. *Journal of Orthopaedic Research*.

[47] Peter C. P., Cook W. O., Nunamaker D. M., Provost M. T., Seedor J. G., Rodan G. A. (1996). Effect of alendronate on fracture healing and bone remodeling in dogs. *Journal of Orthopaedic Research*.

[48] Einhorn T. A. (2010). Can an anti-fracture agent heal fractures?. *Clinical Cases in Mineral and Bone Metabolism*.

[49] Garrison K. R., Shemilt I., Donell S. (2010). Bone morphogenetic protein (BMP) for fracture healing in adults. *Cochrane Database of Systematic Reviews*.

